# Comparison of single posterior debridement, bone grafting and instrumentation with single-stage anterior debridement, bone grafting and posterior instrumentation in the treatment of thoracic and thoracolumbar spinal tuberculosis

**DOI:** 10.1186/s12893-018-0405-4

**Published:** 2018-09-03

**Authors:** Yongchun Zhou, Weiwei Li, Jun Liu, Liqun Gong, Jing Luo

**Affiliations:** 1 Department of Orthopedic, Shaanxi Provincial People’s Hospital, 256# You-yi West Road, Xi’an, 710068 Shaanxi, People’s Republic of China; 2 Department of Nursing administration, Honghui Hospital, Xi’an Jiaotong University College of Medicine, 555# You-yi East Road, Xi’an, 710054 Shaanxi, People’s Republic of China

**Keywords:** Thoracic and thoracolumbar, Spinal tuberculosis, Combined anterior and posterior, Single posterior, Debridement

## Abstract

**Background:**

To compare the clinical efficacy of single posterior debridement, bone grafting and instrumentation with that of single-stage anterior debridement, bone grafting and posterior instrumentation for treatment of adult patients with thoracic and thoracolumbar spinal tuberculosis (TB).

**Methods:**

We performed a retrospective analysis of 64 adult patients with thoracic and thoracolumbar spinal TB who underwent surgery between January 2011 and December 2014. Of the 64 patients, 34 patients were treated using a single posterior-only approach (posterior debridement, bone grafting and instrumentation; Group A). Thirty patients were treated with a combined anterior and posterior approach (single-stage anterior debridement, bone grafting and posterior instrumentation; Group B). Clinical manifestations, laboratory and imaging results were subjected to statistical analysis.

**Results:**

The mean (±standard deviation) duration of follow-up was 16.8 ± 1.4 months (range, 10–34). Bony fusion was achieved in all the bone grafts with no loosening or breakage of internal fixation. In both of the groups, the visual analog scale (VAS) pain score, ESR and CRP at 6 weeks after operation and at the most recent follow-up were significantly lower than the preoperative level (*p* < 0.05). The operation time, intraoperative blood loss and length of hospital stay in group A were significantly less than those in group B (*P* < 0.05). As of most recent follow-up, no significant between-group difference was observed with respect to the American Spinal Injury Association classification status (*p* > 0.05). Furthermore, no significant between-group difference was observed with respect to preoperative kyphosis angle, and postoperative angle correction and angle correction rate (*P* > 0.05). One patient in group A relapsed 20 months after operation, and was successfully treated with debridement using the combined anterior and posterior approach.

**Conclusion:**

Single posterior debridement, bone grafting and instrumentation for treatment of thoracic and thoracolumbar spinal TB can achieve similar curative effect as that with single-stage anterior debridement, bone grafting and posterior instrumentation, and is associated with additional advantages of shorter operation time, less bleeding and shorter length of hospital stay.

## Background

The incidence of spinal tuberculosis (TB) has shown a steady increase in developing countries and the condition often leads to severe kyphosis and permanent paralysis [1, 2]. Chemotherapy is essential for the treatment of spinal TB; however, surgical treatment is often required to improve neurological function and kyphosis. Reasonable debridement, internal fixation and fusion can significantly relieve back pain, improve neurological function, and prevent or correct kyphosis in these patients [3].

The main purpose of surgical treatment is radical debridement, nerve decompression and reconstruction of spine to prevent or improve kyphosis. Currently, there is no clear consensus on the optimal surgical strategy for patients with thoracic and thoracolumbar spinal TB. The anterior approach provides direct access to the site of the lesion, which facilitates the removal of the lesion and reconstruction of the defect. However, the anterior approach does not provide adequate leverage for the correction of kyphosis; moreover, fixation performed through the anterior approach necessitates the ligation of the segmental arteries owing to the risk of massive bleeding [4, 5]. The combined use of anterior and posterior approach in a single-stage surgery serves to overcome the limitations of the anterior-only approach and has been widely adopted with good results [6, 7]. It should be noted that the use of a combined anterior and posterior approach increases the operation time, and is associated with greater trauma and higher risk of complications, not conducive to early rehabilitation of patients [3]. Good results have been reported with use of posterior approach [8, 9]; however, it is associated with inadequate treatment of the lesion in front of the vertebral body.

Thoracic and thoracolumbar segments are the common locations of spinal TB. Due to the small volume of the spinal canal and the poor blood supply of the spinal cord, lower extremity weakness or other neurological deficits are liable to occur in patients with severe bone destruction or instability of the spine [10]. In this study, we explored the clinical outcomes of single posterior debridement, bone grafting and instrumentation and single-stage anterior debridement, bone grafting and posterior instrumentation for treatment of adult patients with thoracic and thoracolumbar spinal TB.

## Methods

### Patient population

We performed a retrospective analysis of 64 patients with active thoracic and thoracolumbar spinal TB (without active TB) treated between January 2011 and December 2014. The diagnosis of active spinal TB was based on clinical symptoms, laboratory investigations [high erythrocyte sedimentation rate (ESR) and C-reactive protein (CRP)], and radiographic examination [x-ray, computed tomography (CT), and magnetic resonance imaging (MRI)]. Moreover, pathological examination was performed to confirm the diagnosis. Other inclusion criteria were: (1) progressive nerve injury; (2) ineffective conservative treatment, and no significant decrease in inflammatory markers after anti-TB treatment; (3) kyphosis (kyphosis angle > 16°); and (4) back pain. The ethics review committee of the Shaanxi Provincial People’s Hospital approved the study protocol. All the patients provided written informed consent for the use and publication of data for research purposes.

### Preoperative preparation

Conventional anti-TB treatment was administered for 2–4 weeks before operation. HREZ anti-tuberculous regimen was adopted: isoniazid (300 mg, oral administration or intravenous drip, once a day), rifampin (450 mg, oral administration, once a day), pyrazinamide (1.5 g, oral administration, once a day), and ethambutol (750 mg, oral administration, once a day). Surgery was performed when the ESR had significantly decreased (≤ 40 mm/h), the temperature had returned to normal, and anemia and hypoproteinemia were resolved completely.

### Operative technique

**Fig. 1:**
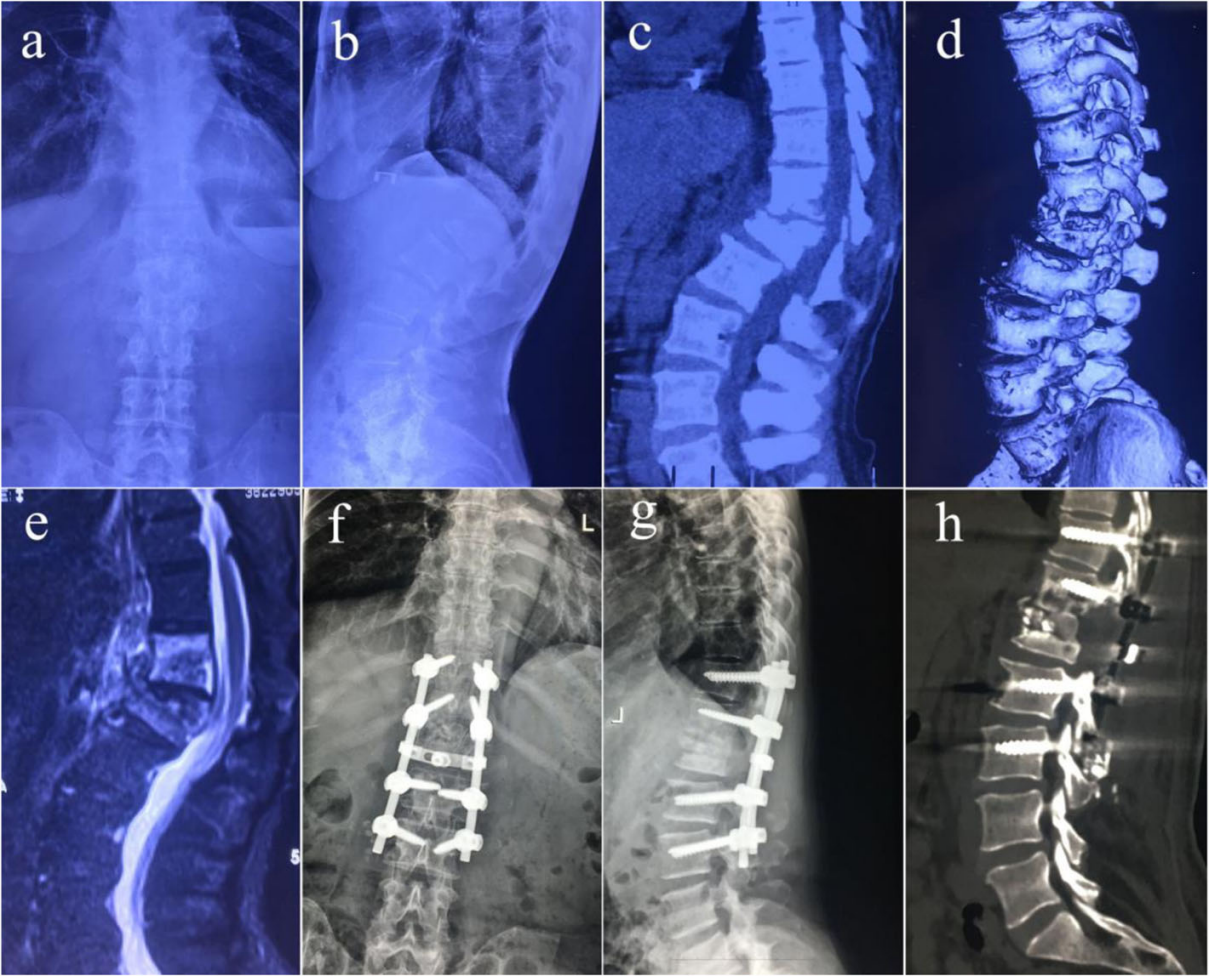
Imaging findings of a 36-year-old adult patient with thoracolumbar spinal TB who underwent single posterior debridement, bone grafting and instrumentation. **a**-**e** Preoperative x-ray, CT and MR images showing destruction of T12 and L1 and a paravertebral abscess. **f**-**h** Postoperative anteroposterior and lateral x-rays, and CT showing fixation of T11-L3, and vertebral height recovery

Patients in group A were placed in a prone position, and pedicle screws were inserted in the normal vertebral body adjacent to the upper and lower affected vertebrae. Internal fixation rod was temporarily installed on the less affected side, followed by the excision of the laminae to protect the spinal dura. From the more severe side of the lesion, the upper and lower segments of the diseased vertebra were removed and the intervertebral space was exposed. Then, the necrotic intervertebral disc, pathological vertebral body and paravertebral abscess were removed, followed by the removal of the lesion on the other side using the same method. After the removal of the lesion, a large amount of saline was used to wash the focal zone. After washing, a strip of bone graft was fixed between the residual vertebral bodies of the diseased vertebra. An autogenous iliac bone graft of appropriate size was closely embedded into the intervertebral bone grafting groove, and the pre-bent bar was installed, followed by the correction of kyphosis with moderate pressure. After confirmation of satisfactory internal fixation and correction of kyphosis deformity by C-arm fluoroscopy, a drainage tube was placed and the incision was closed layer by layer (Fig. 1).

**Fig. 2:**
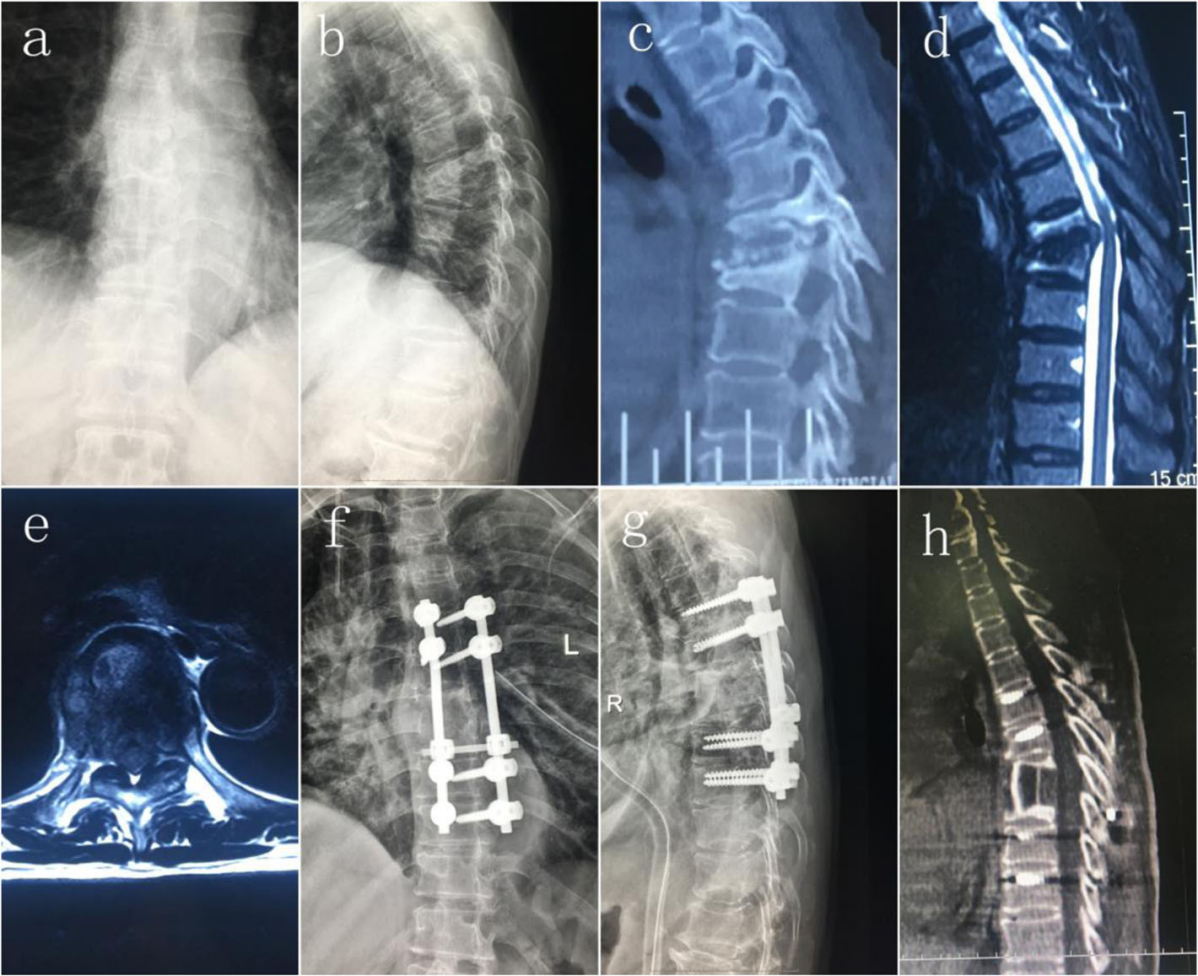
Imaging findings of a 68-year-old patient with thoracic spinal TB who underwent single-stage anterior debridement/bone grafting/posterior instrumentation. **a**-**e** Preoperative anteroposterior and lateral x-rays, CT and MR images showing destruction of T6 and T7 and a paravertebral abscess. **f**-**h** Postoperative anteroposterior and lateral x-rays and CT showing fixation of T4-T9

In group B, the patient was placed in a supine position and the extra pleural or extra peritoneal anterolateral approach was used to avoid pleural or peritoneal injury. After the location of intervertebral space, the collapsed intervertebral disc and the vertebral body were removed along with the paraspinal and psoas abscess. After the complete removal of the lesion, an autologous iliac bone graft or titanium mesh filled with autologous bone was implanted into the bone graft to reconstruct the anterior column. Then, the patients were placed in a prone position, and pedicle screws were inserted in the normal vertebral body adjacent to the upper and lower affected vertebrae. The connecting rod was installed to stabilize the spine. Vertebral compression was determined according to the status of the compression of spinal cord from the posterior spine. Correction of kyphosis was achieved by moderate posterior compression. The incision was sutured after confirmation of internal fixation in good position with C-arm fluoroscopy (Fig. 2).

### Postoperative care

The drainage tube was removed 24–72 h after the operation depending on the drainage volume. Prophylactic antibiotics were used for 3 days postoperatively. Anti-tuberculous therapy was continued after the operation. Pyrazinamide was withdrawn after 6 months of treatment, while the other anti-tuberculous drugs (HRE) were continued for 10–12 months. Non weight-bearing walking aided by braces was recommended 6–8 weeks after operation, while normal weight-bearing activity was allowed only after confirmation of intervertebral fusion on x-ray and CT examinations.

### Evaluation standard

The American Spinal Injury Association (ASIA) scale was used to evaluate preoperative and postoperative spinal cord injury. Bone graft fusion, loss of correction angle and internal fixation failure were evaluated by imaging examination. Visual analog scale (VAS) scores were used to assess the severity of pain. Besides, ESR and CRP levels were assessed to evaluate the disease activity. Moon bone graft fusion method was used to evaluate the bone graft fusion [10]. In addition, preoperative and postoperative kyphosis angle was evaluated using the standard method for the measurement of kyphosis angle [11].

### Statistical analysis

SPSS version 19.0 statistical software (SPSS, Inc., Chicago, IL, USA) was used to process the data. Wilcoxon signed rank test was used to compare preoperative and postoperative ASIA classification. Paired *t* test was used to compare the preoperative and postoperative degree of kyphotic deformity, and ESR and CRP levels. Independent sample *t* test was used to assess the between-group differences with respect to various laboratory and physical parameters. The rank sum test was used for the analysis of discrepancy in normal distribution. *P* < 0.05 was considered indicative of significant difference.

## Results

Out of the 64 patients, 34 patients [25 male and 9 female; mean age ± standard deviation (SD): 39.4 ± 12.0 years (range, 18–70)] were included in group A (treated with posterior-only approach for debridement, bone grafting and instrumentation), while 30 patients [22 male and 8 female; mean age: 40.6 ± 12.5 years (range, 19–71)] were included in group B (treated with combined anterior and posterior approach in a single surgery). All the patients underwent x-ray examination (CT examination in some patients) and showed bone graft fusion. None of the patients showed bone nonunion, false joint formation, internal fixation loosening, or fracture as of the most recent follow-up. The mean time of fusion in groups A and B were 8.6 ± 0.4 months (range, 6–12) and 8.4 ± 0.5 months (range, 5–12), respectively; the between-group difference was not statistically significant (*P >* 0.05). The operation time, volume of intraoperative blood loss, and the length of hospital stay were more favorable in group A as compared to that in group B (*P* < 0.05). Tables 1 and 2 list patient information, surgical details and postoperative characteristics.

**Table 1:** Patient and surgical characteristics and outcomes of the two groups

Characteristic	Group A (*n* = 34)	Group B (*n* = 30)
Sex (male)	25 (73.5%)	22 (73.3%)
Age at initial operation (years)	39.4 ± 12.0 (18–70)	40.6 ± 12.5(19–71)
Operation time (min)^*^	160.4 ± 20.6(116–253)	231.5 ± 27.4 (164–312)
Intraoperative bleeding (ml)^*^	760.7 ± 146.2 (560–1100)	1023.8 ± 197.9 (753–1350)
Time to abscess disappearance (postoperative months)	8.1 ± 0.5 (7–12)	8.0 ± 0.6 (6–12)
Time to bone fusion (postoperative months)	8.6 ± 0.4 (6–12)	8.4 ± 0.5 (5–12)
Hospitalization day (days)*	23.3 ± 4.5(20–29)	26.5 ± 3.5(22–35)

Data are presented as *n* (%) or mean ± standard deviation (range)

^*^P < 0.05. Comparison between group A and group B

**Table 2:** Distributions of lesions sites involved thoracic and thoracolumar spinal tuberculosis

Group	Lesion sites							
	T6–7	T7–8	T8–9	T10–11	T11–12	T12-L1	T12-L1	L1–2
A (*n* = 34)	3	4	3	5	6	6	4	3
B (*n* = 30)	3	3	3	4	5	5	4	3

The mean duration of follow-up in the entire study population was 16.8 ± 1.4 months (range, 10–34). In group A, superficial wound infection occurred in 1 patient, which healed after antibiotic treatment. In group B, 2 patients developed postoperative superficial skin infection, which was cured anti-infection treatment. Besides, 1 case of intercostal neuralgia was relieved after symptomatic treatment. Furthermore, 1 patient developed wound dehiscence 20 months after operation, which was confirmed to be TB relapse by imaging examination, and was treated with debridement using a combined anterior and posterior approach. None of the patients in group B experienced relapse of TB. No serious neurological complications occurred in any of the groups.

Table 3 summarizes the changes in the VAS score, ESR and CRP levels 6 weeks after operation and at the most recent follow-up. The VAS score, ESR, and CRP level at 6 weeks after operation and at the most recent follow-up were significantly lower than the preoperative levels in both groups (*P* < 0.05). Furthermore, VAS score, ESR and CRP at 6 weeks after operation were significantly higher than those at the most recent follow-up (*P* < 0.05).

**Table 3:** Measures of surgical outcomes of the two groups

Measure	VAS			CRP (mg/L)			ESR (mm/h)		
	Pre-op	6 weeks post-op	Final follow-up	Pre-op	6 weeks post-op	Final follow-up	Pre-op	6 weeks post-op	Final follow-up
Group A	5.9 ± 0.8	3.5 ± 0.8^*^	2.4 ± 0.7^Δ^	18.5 ± 4.5	8.1 ± 1.2^*^	3.0 ± 0.6^Δ^	39.2 ± 8.2	22.1 ± 1.5^*^	9.5 ± 1.0^Δ^
Group B	5.6 ± 0.9	3.4 ± 0.8^*^	2.3 ± 0.7^Δ^	18.2 ± 5.1	8.0 ± 1.1^*^	2.9 ± 0.5^Δ^	39.3 ± 8.3	21.8 ± 1.4^*^	9.4 ± 1.1^Δ^

*VAS* Visual Analogue Scale, *ESR* Erythrocyte Sedimentation Rate, *CRP* C-Reactive Protein, *Pre-op* Preoperative, *Post-op* Postoperative

*P < 0.05 vs. preoperative

^Δ^*P* < 0.05 vs. 6 weeks postoperative

Table 4 shows the changes in ASIA classification in the two groups. In group A, out of the 30 patients with preoperative neural deficit, 27 patients showed complete postoperative recovery. Out of the 28 patients with preoperative nerve dysfunction in group B, 25 patients recovered to normal postoperatively. As of the most recent follow-up, no significant between-group difference was observed with respect to improvement in ASIA classification status (*P* > 0.05).

**Table 4:** Neurological recovery according to Frankel grade

Time point	Group A					Group B				
	A	B	C	D	E	A	B	C	D	E
Preoperative		1	3	26	4			2	26	2
Final follow-up*				3	31				3	27

H_C-A_ = 41.3, H_C-B_ = 16.8, H_CA-B_ = 0.3;

*P < 0.05 vs. preoperative

Table 5 presents data related to kyphosis correction and kyphosis loss. There was no significant between-group difference with respect to preoperative kyphosis angle (*P* > 0.05); besides, there was no significant between-group difference with respect to postoperative angle correction and angle correction rate (*P* > 0.05). Furthermore, the kyphosis angle loss and kyphosis loss rate did not differ between the two groups as of the most recent follow-up (*P* > 0.05). The kyphosis angle after operation and at the most recent follow-up were significantly lower than the respective preoperative kyphosis angle in both groups (*P* < 0.05).

**Table 5:** Kyphosis correction and kyphosis lost in two groups

Group	Pre-operative kyphosis angle(°)^*^	Post-operation			Final follow-up		
		Kyphosis Angle (°)^Δ^	Angle Correction (°)^†^	Correction Rate(%)^▲^	Kyphosis Angle (°)	Angle lost (°)^□^	Lost rate(%)^#^
A	26.1 ± 6.0	9.9 ± 3.7	16.4 ± 5.6	62.4 ± 12.1	11.2 ± 3.3	1.4 ± 1.1	5.5 ± 5.8
B	23.7 ± 3.7	9.6 ± 3.3	14.2 ± 4.1	59.3 ± 13.0	11.0 ± 3.2	1.0 ± 0.9	5.3 ± 4.6

^*^One-way analysis of variance, compared pre-operative kyphosis angle between two groups, *P* > 0.05

^Δ^One-way analysis of variance, compared kyphosis angle with pre-operative in two groups, *P*_A_ < 0.05, *P*_B_ < 0.05

^†^One-way analysis of variance, compared angle correction between two groups, *P* > 0.05

^▲^One-way analysis of variance, compared correction rate between two groups, *P* > 0.05

^□^One-way analysis of variance, compared angle lost between two groups, *P* > 0.05

^#^One-way analysis of variance, compared angle lost rate between two groups, *P* > 0.05

## Discussion

Spinal TB most commonly affects the thoracic vertebrae and the thoracolumbar spine. The latter represents the area of transition from a relatively fixed thoracic vertebra and a relatively mobile lumbar spine; therefore, the risk of back pain and paraplegia is greater once the disease occurs [12, 13]. In clinical practice, patients with thoracolumbar TB often show severe bone destruction, spinal cord compression and/or kyphotic deformity. Therefore, conservative treatment alone is often fails to relieve spinal cord compression, improve nerve dysfunction and prevent spinal deformity, while surgery is often the preferred treatment modality [14]. The purpose of the surgical treatment of thoracic and thoracolumbar spinal TB is to remove the lesion thoroughly, relieve spinal cord compression and to reconstruct the spine.

Anti-TB drug therapy is an essential measure for the treatment of spinal TB and also the basis of surgical treatment. Without regular anti-TB treatment, surgery alone is extremely dangerous and ineffective. Effective surgical treatment can be performed only under effective anti-TB treatment. In this study, with the exception of 2 patients who showed progressive neurological impairment, all the patients were treated with anti-TB drugs for over 2 weeks prior to the operation. Furthermore, surgical treatment was performed only when ESR was < 40 mm/h, and hemoglobin level was at least 100 g/L.

Parthasarathy et al. [15] conducted a retrospective analysis of 235 cases of spinal TB treated with conservative and surgical treatment; the cure rates of spinal TB with isoniazid alone, and combination chemotherapy with rifampin were not worse than those achieved with surgery. However, the authors recommended active surgical treatment for patients who develop neurological deterioration or progressive kyphosis during the course of anti-TB chemotherapy. In this setting, not only does surgical treatment aim to remove TB lesions, but it also aims to relieve spinal cord compression, correct kyphosis and reconstruct the spine. From this point of view, it is appropriate to apply internal fixation in thoracic and thoracolumbar spinal TB operations [16]. Single-stage anterior debridement, bone grafting and instrumentation have been considered as the gold standard for treatment of spinal TB [17]. However, this method often achieves inadequate fixation rigidity and orthopedic strength and is associated with a high risk of vascular injury due to poor access to the site of lesion [18, 19]. Since the combined anterior and posterior approach overcomes the limitations of poor stability and correction associated with the single anterior approach, it has been used widely. However, the combined approach is associated with excessive intraoperative bleeding, prolonged operation time, increased length of hospital stage, and a relatively high risk of complications [6, 7, 20, 21]. Wang et al. [20] evaluated 28 patients with thoracolumbar TB who underwent surgery using the combined anterior and posterior approach. They reported 90.4% correction of the kyphosis angle; however, compared to that with single posterior approach, the intraoperative bleeding, operation time and length of hospital stay were relatively greater. Moreover, they reported wound infection, sinus formation at the drainage tube and other surgical complications. Laheri et al. [21] reported 62.5% correction rate of kyphosis in their series of 38 patients with spinal TB who underwent surgery using the combined anterior and posterior approach. In the present study, all the patients in group B achieved 59.3% correction of the kyphosis angle, and the volume of intraoperative bleeding, operation time and length of stay were also greater than that in group A (single posterior approach). Besides, 3 patients in group B experienced surgical complications, as against 1 patient only in group A.

With the development of surgical techniques for spinal TB, single posterior debridement, bone grafting and instrumentation can achieve correction of kyphosis deformity and spinal stabilization. Zhang et al. reported good clinical outcomes in patients with thoracic vertebral TB [22]. Zhou et al. [3] reported good surgical results with single posterior approach for treatment of lumbar TB. In the present study, 34 patients in group A received single posterior debridement, bone grafting and instrumentation for the treatment of thoracic and thoracolumbar spinal TB. Postoperative ASIA classification showed significant improvement, accompanied by significant decrease in postoperative Cobb angle and ESR level (*P* < 0.05 vs. preoperative level), which suggests good efficacy of surgical treatment. No significant difference was observed with respect to the VAS score, ASIA grading, improvement in Cobb angle or ESR level when compared with those achieved with the combined anterior and posterior approach. However, due to poor exposure to the anterior structure of the spine, the single posterior approach is not suitable for patients with large paravertebral abscess. In this study, there was 1 patient with lumbar vertebral TB with paravertebral abscess in the single posterior approach group. Due to incomplete removal of the lesions, the large paravertebral abscess appeared again 20 months after operation in spite of the anti-tuberculous treatment, which exemplifies this shortcoming.

In this study, patients with spinal TB treated by posterior approach experienced obvious relief of back pain after operation. The intervertebral bone graft fusion was reliable, and there was less long-term kyphosis loss and low recurrence rate. However, it is necessary to evaluate the indications for use of this approach carefully, and due discretion should be exercised before use of posterior approach in patients with thoracic and thoracolumbar spinal TB with large paravertebral abscess. We believe that the rationale for use of the posterior approach is it allows for the removal of sclerotic bone around the lesion. Simultaneously, a small amount of residual TB-like lesion and pus can resolve with standard anti-TB chemotherapy after operation. Therefore, the importance of adequate anti-TB treatment after operation can hardly be overemphasized. Moreover, operations of “radical scavenging” are relative and not absolute. TB recurrence is caused mostly by drug-resistant Mycobacterium TB or postoperative chemotherapy with nonstandard regimen [23]. This study also has certain limitations, including the relatively small sample size and the duration of follow-up.

## Conclusion

Single posterior debridement, bone grafting and instrumentation for treatment of thoracic and thoracolumbar spinal TB can achieve similar curative effect as that achieved with single-stage anterior debridement, bone grafting and posterior instrumentation, but with additional advantages of shorter operation time, less bleeding and shorter length of hospital stay. To sum up, single posterior approach is a favorable method for the treatment of thoracic and thoracolumbar spinal TB.

## Abbreviations

ASIA: American Spinal Injury Association
CRP: C-reactive protein
CT: Computed tomography
ESR: Erythrocyte sedimentation rate
MRI: Magnetic resonance imaging
Post-op: Post-operation
Pre-op: Pre-operation
TB: Tuberculosis
VAS: Visual analog scale
